# Computation of elementary modes: a unifying framework and the new binary approach

**DOI:** 10.1186/1471-2105-5-175

**Published:** 2004-11-04

**Authors:** Julien Gagneur, Steffen Klamt

**Affiliations:** 1 Cellzome AG, Meyerhofstr. 1, 69117 Heidelberg, Germany; 2 Max Planck Institute for Dynamics of Complex Technical Systems, Sandtorstr. 1, D-39106 Magdeburg, Germany

## Abstract

**Background:**

Metabolic pathway analysis has been recognized as a central approach to the structural analysis of metabolic networks. The concept of elementary (flux) modes provides a rigorous formalism to describe and assess pathways and has proven to be valuable for many applications. However, computing elementary modes is a hard computational task. In recent years we assisted in a multiplication of algorithms dedicated to it. We require a summarizing point of view and a continued improvement of the current methods.

**Results:**

We show that computing the set of elementary modes is equivalent to computing the set of extreme rays of a convex cone. This standard mathematical representation provides a unified framework that encompasses the most prominent algorithmic methods that compute elementary modes and allows a clear comparison between them. Taking lessons from this benchmark, we here introduce a new method, the binary approach, which computes the elementary modes as binary patterns of participating reactions from which the respective stoichiometric coefficients can be computed in a post-processing step. We implemented the binary approach in FluxAnalyzer 5.1, a software that is free for academics. The binary approach decreases the memory demand up to 96% without loss of speed giving the most efficient method available for computing elementary modes to date.

**Conclusions:**

The equivalence between elementary modes and extreme ray computations offers opportunities for employing tools from polyhedral computation for metabolic pathway analysis. The new binary approach introduced herein was derived from this general theoretical framework and facilitates the computation of elementary modes in considerably larger networks.

## Background

The background section presents the importance of computing elementary modes for metabolic system analysis, its computational difficulties and the existence of various known algorithms. A theoretical section brings these algorithms into a unified framework. In a following section we introduce a new approach, called the binary approach. Although relying on concepts introduced in the theoretical section, this section gives enough practical details to be stand-alone for the implementer. Results obtained from example networks and a conclusion section close the article.

### Definition and benefits of elementary modes

We consider a metabolic network with *m *metabolites and *q *reactions. Reactions may involve further metabolites that are not considered as proper members of the system of study. The latter metabolites, considered to be buffered, are called *external *metabolites in opposition to the *m *metabolites within the boundary of the system, called *internal *metabolites. The stoichiometry matrix **N **is an *m *× *q *matrix whose element *n*_*ij *_is the signed stoichiometric coefficient of metabolite *i *in reaction *j *with the following sign convention: negative for educts, positive for products. Some reactions, called irreversible reactions, are thermodynamically feasible in only one direction under the normal conditions of the system. Therefore, reaction indices are split into two sets: *Irrev *(the set of irreversible reaction indices) and *Rev *(the set of reversible reaction indices). A flux vector (flux distribution), denoted **v**, is a *q*-vector of the reaction space ^*q*^, in which each element *v*_***i ***_describes the net rate of the *i*^**th **^reaction. Sometimes we are interested only in the relative proportions of fluxes in a flux vector. In this sense, two flux vectors **v **and **v' **can be seen to be equivalent, denoted by **v **≃ **v'**, if and only if there is some *α *> 0 such that **v **= *α *· **v'**.

Metabolism involves fast reactions and high turnover of substances compared to events of gene regulation. Therefore, it is often assumed that metabolite concentrations and reaction rates are equilibrated, thus constant, in the timescale of study. The metabolic system is then considered to be in quasi steady state. This assumption implies **Nv **= **0**. Thermodynamics impose the rate of each irreversible reaction to be nonnegative. Consequently the set of feasible flux vectors is restricted to

*P *= {**v **∈ ^*q *^: **Nv **= **0 **and *v*_*i *_≥ 0, *i *∈ *Irrev*}     (1)

*P *is a set of *q*-vectors that obey a finite set of homogeneous linear equalities and inequalities, namely the |*Irrev*| inequalities defined by *v*_*i *_≥ 0, *i *∈ *Irrev *and the *m *equalities defined by **Nv **= **0**. *P *is therefore – by definition – a convex polyhedral cone [1].

Metabolic pathway analysis [2-5] serves to describe the (infinite) set *P *of feasible states by providing a (finite) set of vectors that allow the generation of any vectors of *P *and are of fundamental importance for the overall capabilities of the metabolic system. One of this set is the so-called set of elementary (flux) modes (EMs). For a given flux vector **v**, we note *R*(**v**) = {*i *: *v*_*i *_≠ 0} the set of indices of the reactions participating in **v**. Hence, *R*(**v**) can be seen as the underlying pathway of **v**. By definition, a flux vector **e **is an *elementary mode *(EM) if and only if it fulfills the following three conditions [6,7]:



In other words, **e **is an EM if and only if it works at quasi steady state, is thermodynamically feasible and there is no other non-null flux vector (up to a scaling) that both satisfies these constraints and involves a proper subset of its participating reactions. Note that with this convention, reversible modes are here considered as two vectors of opposite directions.

The concept of elementary modes (and, with some restrictions, the very similar concept of extreme pathways [8-10]) has proven useful in many ways and has become an important theoretical tool for systems biology as well as for biotechnology and metabolic engineering (see review [5]). Because the metabolic network structure becomes now available at a genome-scale for an increasing number of microorganisms, this approach is well-suited to today's metabolic studies. Here, we give a short overview on the major applications and variants:

(1) *Identification of pathways*: The set of EMs comprises all admissible routes through the network and thus of "pathways" in the classical sense, i.e. of routes that convert some educts into some products [5].

(2) *Network flexibility*: The number of EMs is at least a rough measure of the network's flexibility (redundancy, structural robustness) to perform a certain function [11-13].

(3) *Identification of all pathways with optimal yield*: Consider the linear optimization problem, where all flux vectors with optimal product yield are to be identified, i.e. where the moles of products generated per mole of educts is maximal. Then, one or several of the EMs reach this optimum and any optimal flux vector is a convex combination of these optimal EMs [3,14].

(4) *Importance of reactions*: The importance or relevance of a reaction can be assessed by its participation frequency or/and flux values in the EMs.

(4a) *Inference of viability of mutants*: If a reaction is involved in all growth-related EMs its deletion can be predicted to be lethal, since all EMs would disappear [11].

(4b) A more quantitative measure of the importance of a reaction has been given by "control-effective fluxes" (CEFs, [11]). The CEFs take also the efficiency of each mode as well as the absolute flux values of the respective reaction in the EMs into account. CEFs have been used to predict transcript ratios [11,15].

(5) *Reaction correlations*: EMs can be used to analyze structural couplings between reactions, which might give hints for underlying regulatory circuits [14,16,17]. An extreme case is an enzyme (or reaction) subset (set of reactions which can operate only together) or a pair of mutually excluding reactions (two reactions never occurring together in any EM [10]).

(6) *Detection of thermodynamically infeasible cycles*: EMs representing internal cycles (without participation of external material or energy sources) are infeasible by laws of thermodynamics and thus reflect structural inconsistencies [18,19].

(7) The framework of pathway analysis also allows us to combine and to study stoichiometric constraints together with *regulatory rules *[20].

(8) *Minimal cut sets: *EMs allow for a computation of minimal cut sets that represent minimal cuts (deletion sets) in the network repressing certain metabolic functions [21].

(9) The *α-spectrum *has been introduced to quantify the involvement of extreme pathways in a particular flux distribution (e.g. from an experiment) [22]. Since the decomposition of a flux vector into extreme pathways is usually not unique, the *α-spectrum *specifies a range of possible weights for each extreme pathway. The same could be defined for EMs.

### Computational limitations and algorithm variants

Due to the combinatorial explosion in the number of EMs in large networks [23], computing EMs is known to be a hard computational task, so far restricting elementary-mode analysis to medium-scale networks. Several algorithms (and derivations thereof) have been developed for computing EMs. The two most prominent ones are the algorithm elaborated by Schuster et al. [4] and the recently developed null-space approach by Wagner [24]. The latter considerably accelerated the computation speed and thus shifted the current limitation – at least for a typical PC – from computation time to the memory requirement.

Here we show that both the Schuster algorithm as well as that by Wagner can be embedded in a more general algorithmic framework stemming originally from computational geometry. These studies do not only give a summarizing point of view, they also lead to a crucial modification of the existing algorithms, decreasing the required memory for computing and storing EMs drastically.

## Results

### A unified framework

#### Elementary modes as extreme rays in networks of irreversible reactions

In the particular case of a metabolic system with only irreversible reactions, the set of admissible reactions reads:

*P *= {**v **∈ ^*q *^: **Nv **= **0 **and **v **≥ **0**}     (3)

**Figure 1:**
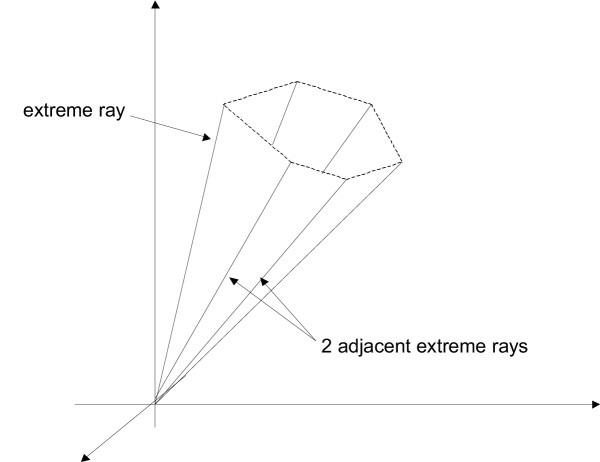
A pointed polyhedral cone. Dashed lines represent virtual cuts of unbounded areas

Compared with (1) *P *is in this case a particular, namely a *pointed *polyhedral cone (an example is depicted in Figure 1). This geometry can be intuitively understood, noting that there are certainly 'enough' intersecting half-spaces (given by the inequalities **v **≥ **0**) to have this 'pointed' effect in **0**: *P *contains no real line (otherwise there coexist **x **and **-x **not null in *P*, a contradiction with the constraint **v **≥ **0**). The figure even suggests that a pointed polyhedral cone can be either defined in an implicit way, by the set of constraints as we did until now, or in an explicit or generative way, by its 'edges', the so-called *extreme rays *(or *generating vectors*) that unambiguously define its boundaries. In the following, we show that elementary modes always correspond to extreme rays of a particular pointed cone as defined in (3) and that their computation therefore matches to the so-called extreme ray enumeration problem, i.e. the problem of enumerating all extreme rays of a pointed polyhedral cone defined by its constraints. An overview on general and current issues on extreme ray enumeration can be found in [25]. For the sake of consistency, we use this reference as a main source and adopt the same mathematical notations.

The pointed polyhedral cone is the central mathematical object throughout this work; therefore we shall introduce more precise definitions and results surrounding it.

*P *is a *pointed polyhedral cone *of ^*d *^if and only if *P *is defined by a full rank *h *× *d *matrix **A **(*rank*(**A**) = *d*) such that,

*P *= *P*(**A**) = {**x **∈ ^*d *^: **Ax **≥ **0**}     (4)

The *h *rows of the matrix **A **represent *h *linear inequalities, whereas the full rank mention imposes the "pointed" effect in **0**. Note that a pointed polyhedral cone is, in general, not restricted to be located completely in the positive orthant as in (3). For example, the cone considered in extreme-pathway analysis may have negative parts (namely for exchange reactions), however, by using a particular configuration it is ensured that the spanned cone is pointed [8].

Now we must characterize the extreme rays. A vector **r **is said to be a *ray *of *P*(**A**) if **r **≠ **0 **and for all *α *> 0, *α *· **r **∈ *P*(**A**). We identify two rays **r **and **r' **if there is some *α *> 0 such that **r **= *α *· **r' **and we denote **r **≃ **r'**, analogous as introduced above for flux vectors. For any vector **x **in *P*(**A**), the *zero set *or active set *Z*(**x**) is the set of inequality indices satisfied by **x **with equality. Noting **A**_*i*• _the *i*^**th **^row of **A**, *Z*(**x**) = {*i *: **A**_*i*•_**x **= 0}. Zero sets can be used to characterize extreme rays. For simplicity, we adopt in this document the following characteristic ([25] for example) as a working definition of extreme rays.

Definition 1: Extreme ray

*Let ***r ***be a ray of the pointed polyhedral cone *P(**A**). *The following statements are equivalent*:

(*a*) **r ***is an extreme ray of *P(**A**)

(b) if **r' **is a ray of P(**A**) with *Z*(**r**) ⊆ *Z*(**r'**) then **r' **≃ **r**

Since **A **is full rank, **0 **is the unique vector that solves all constraints with equality. The extreme rays are those rays of *P*(**A**) that solve a maximum but not all constraints with equalities. This is expressed in (b) by requiring that no other ray in *P*(**A**) solves the same constraints plus additional ones with equalities. Note that in (b) *Z*(**r**) = *Z*(**r'**) consequently holds.

An important property of the extreme rays is that they form a finite set of generating vectors of the pointed cone ([25] for example): any vector of *P*(**A**) can be expressed as a non-negative linear combination of extreme rays, and the converse is true: all non-negative combinations of extreme rays lie in *P*(**A**). Moreover, the set of extreme rays is the unique minimal set of generating vectors of a pointed cone *P*(**A**) (up to positive scalings).

Lemma 1: EMs in networks of irreversible reactions

In a metabolic system where all reactions are irreversible, the EMs are exactly the extreme rays of *P *= {**v **∈ ^*q *^: **Nv **= **0 **and **v **≥ **0**}.

**Proof**: *P *is the solution set of the linear inequalities defined by  where **I **is the *q *× *q *identity matrix. Since it contains **I**, **A **is full rank and therefore *P *is a pointed polyhedral cone. All **v **∈ *P *obey **Nv **= **0**, thus the 2*m *first inequalities defined by **A **hold with equality for all vectors in *P *and the inclusion condition of Definition 1 can be restricted to the last *q *inequalities, i.e. the inequalities corresponding to the reactions. Inclusion over the zero set can be equivalently seen as containment over the set of non-zeros in **v**, i.e. *R*(**v**). Consequently, **e **∈ *P *is an extreme ray of *P *if and only if: for all **e' **∈ *P *: *R*(**e'**) ⊆ *R*(**e**) ⇒ **e' **= **0 **or **e' **≃ **e**, i.e. if and only if **e **is elementary. Thus, all three conditions in (2) are fulfilled.

#### The general case

In the general case, some reactions of the metabolic system can be reversible. Consequently, **A **does not contain the identity matrix and *P *(as given in (1)) is not ensured to be a pointed polyhedral cone anymore [7]. Because they contain a linear subspace, non-pointed polyhedral cones cannot be represented properly by a unique set of generating vectors composed of extreme rays, albeit a set of generating vectors exists, sometimes also called convex basis [7]. One way to obtain a pointed polyhedral cone from (1) is to split up each reversible reaction into two opposite irreversible ones. Note that this operation is completely analogous to a transformation step used in linear programming to obtain a linear optimization problem in canonical form: free variables v are also split into two variables v^+ ^and v^- ^with v = v^+ ^- v^- ^and v^+^, v^- ^≥ 0 [26]. It has been noticed earlier that this virtual split does not change essentially the outcome: the EMs in the reconfigured network are practically equivalent to the EMs from the original network [10]. Here we prove and precisely characterize this result.

We first introduce some notations. We denote the original reaction network by *S *and the reconfigured network (with all reversible reactions split up) by *S'*. The reactions of *S *are indexed from 1 to *q*. Remember that *Irrev *denotes the set of irreversible reaction indices and *Rev *the reversible ones. An irreversible reaction indexed *i *gives rise to a reaction of *S' *indexed *i*. A reversible reaction indexed *i *gives rise to two opposite reactions of *S' *indexed by the pairs (*i*,+1) and (*i*,-1) for the forward and the backward respectively. The *reconfiguration *of a flux vector **v **∈ ^*q *^of *S *is a flux vector **v' **∈ ^*Irrev *∪ *Rev *× {-1;+1} ^of *S' *such that



Let **N' **be the stoichiometry matrix of *S'*. **N' **can be written as **N' **= [**N **- **N**_*Rev*_] where **N**_*Rev *_consists of all columns of **N **corresponding to reversible reactions. Note that if **v **is a flux vector of *S *and **v' **is its reconfiguration then **Nv **= **N'v'**.

If possible, i.e. if **v' **∈ ^*Irrev *∪ Re*v *× {-1;+1} ^is such that for any reversible reaction index *i *∈ *Rev *at least one of the two coefficients *v'*_(*i*,+1) _or *v'*_(*i*,-1) _equals zero, then we define the reverse operation, called *back-configuration *that maps **v' **back to a flux vector **v **such that:



Theorem 1: EMs in original and in reconfigured networks

Let S be a metabolic system and S' its reconfiguration by splitting up reversible reactions. Then the set of EMs of S' is the union of

a) the set of reconfigured EMs of S

b) *the set of two-cycles made of a forward and a backward reaction of *S' *derived from the same reversible reaction of S*

**Proof**: see Methods.

Thus, the set of EMs of the original network is equivalent (up to the two-cycles) to the set of EMs in the reconfigured network and therefore can be seen as a reduced set of extreme rays of the pointed convex polyhedron as defined by:

*P *= {**v' **∈ ^*q *+ |*Rev*| ^: **N'v' **= **0 **and **v' **≥ **0**}     (5)

Hence, EMs computation can be derived from any extreme ray enumeration algorithm applied to the reconfigured network and followed by vector back-configuration and the elimination of meaningless vectors, namely the two-cycles.

Note that exactly the same procedure – splitting reversible reactions into two irreversible ones – was carried out also in the original work of Clarke [27] on stability analyses in stoichiometric networks. Clarke called the extreme rays of the corresponding cone (5) *extreme currents*. Thus, extreme currents are identical to the EMs in the reconfigured network and, hence, also (up to the 2-cycles) equivalent to the EMs from the original network

#### All known algorithms for computing EMs are variants of the Double Description Method

In the following we present a simple yet efficient algorithm for extreme ray enumeration, the so-called Double Description Method [28]. We show that it serves as a common framework to the most prominent EM computation methods. To reach this generality, we concentrate on mathematical operations regardless to the actual data-structures used in the implementation. Therefore we manipulate objects such as matrices, vectors or inequalities and leave their implementation into tableaus, arrays and so on to the next section.

A *generating matrix ***R **of a pointed polyhedral cone *P*(**A**) is a matrix such that *P*(**A**) = {**x **∈ ^*d *^: **x **= **Rλ **for some **λ **≥ 0}. The pair (**A**,**R**) is called a *Double Description pair*, or DD pair. As mentioned above, the extreme rays form the unique set of minimal generating vectors of *P*(**A**) and thus, considered as set of *d*-vectors, the extreme rays of *P*(**A**) form the columns of a generating matrix **R **that is minimal in terms of number of columns. The pair (**A**,**R**) is then called a *minimal DD pair*.

The strategy of the Double Description Method is to iteratively build a minimal DD pair (**A**_*k*_, **R**_*k*_) from a minimal DD pair (**A**_*k *- 1_, **R**_*k *- 1_), where **A**_*k *_is a submatrix of **A **made of *k *rows of **A**. At each step the columns of **R**_*k *_are the extreme rays of *P*(**A**_*k*_), the convex polyhedron defined by the linear inequalities **A**_*k*_. The incremental step introduces a constraint of **A **that is not yet satisfied by all computed extreme rays. Some extreme rays are kept, some are discarded and new ones are generated. The generation of new extreme rays relies on the notion of *adjacent extreme rays*. Here again, for the sake of simplicity, we adopt a characteristic ([25] for example) as a working definition of adjacent extreme rays.

Definition 2: Adjacent extreme rays

*Let ***r ***and ***r' ***be distinct rays of the pointed polyhedral cone *P(**A**). *Then the following statements are equivalent*:

(a) **r **and **r' **are adjacent extreme rays

(b) if **r" **is a ray of P(**A**) with *Z*(**r**) ∩ *Z*(**r'**) ⊆ *Z*(**r"**) then either **r" **≃ **r **or **r" **≃ **r'**

##### Initialization

The initialization of the double description method must be done with a minimal DD pair. One possibility is the following. Since *P *is pointed, **A **has full rank and contains a nonsingular submatrix of order *d *denoted by **A**_*d*_. Hence, (**A**_*d*_, **A**_*d*_^-1^) is a minimal DD pair which works as initialization and leads directly to step *k *= *d*. Note that there is some freedom in choosing a submatrix **A**_*d *_or some alternative starting minimal DD pair.

##### Incremental step

**Figure 2:**
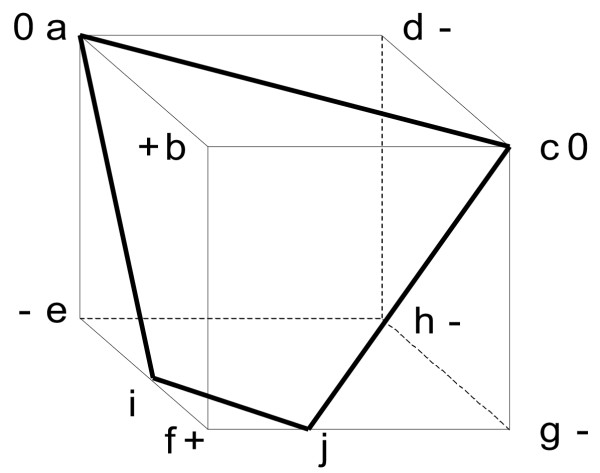
**Double description incremental step**. The scene is best visualized with a polytope; consider the cube pictured here as a ^3 ^projection of a ^4 ^polyhedral cone. Extreme rays from the previous iteration are {a,b,c,d,e,f,g,h} whose adjacencies are represented by edges. For the considered constraint, whose null space is the hyperplane depicted by the bold black border lines, b and f are positive rays, a and c are zero rays, d, e, g and h are negative rays. b, f, a and c satisfy the constraint and are kept for the next iteration. {f,e} and {f,g} are the only two pairs of adjacent positive/negative rays and only they give rise to new rays: i and j at the intersection of the hyperplane and the respective edges. The new polytope is then defined by its extreme rays: {a,b,c,f,i,j}.

Assume (**A**_*k *- 1_, **R**_*k *- 1_) is a minimal DD pair and consider a *k*^**th **^constraint defined by a not yet extracted row of **A**, denoted **A**_*i*•_. Let *J *be the set of column indices of **R**_*k *- 1 _and **r**^*j*^, *j *∈ *J*, its column vectors, i.e. the extreme rays of *P*(**A**_*k *- 1_), the polyhedral cone of the previous iteration. **A**_*i*• _splits *J *in three parts (Figure 2) whether **r**^*j *^satisfies the constraint with strict inequality (*positive *ray), with equality (*zero *ray) or does not satisfy it (*negative *ray):

*J*^+ ^= {*j *∈ *J *: **A**_*i*•_**r**^*j *^> 0}

*J*^0 ^= {*j *∈ *J *: **A**_*i*•_**r**^*j *^= 0}     (6)

*J*^- ^= {*j *∈ *J *: **A**_*i*•_**r**^*j *^< 0}

Minimality of **R**_*k *_is ensured in considering all positive rays, all zero rays and new rays obtained as combination of a positive and a negative ray that are adjacent to each other [25]. For convenience, we denote by *Adj *the index set of the newly generated rays in which every new ray is expressed by a pair of indices corresponding to the two adjacent rays combined. Hence, **R**_*k *_is defined as the set of column vectors **r**^*j*^, *j *∈ *J' *with



The incremental step is repeated until *k *= *h *i.e. having treated all rows of the matrix **A**. The columns of the final matrix **R**_*m *_are the extreme rays of *P*(**A**).

#### Computing EMs

The Double Description Method together with Theorem 1 offers a framework for computing EMs. The only steps to include are a reconfiguration step that splits reversible reactions and builds the matrix **A**, and a post-processing step that gets rid of futile two-cycles and computes the back-configuration. The dimension of the space is given by the number of reactions in the reconfigured network: *q*' = *q *+ |*Rev*|. This results in the general algorithmic scheme as given in Table 1 (from here, all variables for the reconfigured network are written without prime):

**Table 1:** General double description method for EM computation.

**N **← reconfigured stoichiometry matrix [**N **- **N**_*Rev*_] **A **← [**N**^*T *^-**N**^*T *^**I**]^*T*^	**Reconfiguration**
**A**_*q *_← *q *independent rows of **A** **R **← **A**_*q*_^-1^	**Initialization**
for each unprocessed row **A**_*i*• _of **A **do * J*^+ ^← {*j *∈ *J *: **A**_*i*•_**r**^*j *^> 0} * J*^0 ^← {*j *∈ *J *: **A**_*i*•_**r**^*j *^= 0}* * * J*^- ^← {*j *∈ *J *: **A**_*i*•_**r**^*j *^< 0}* * *** *R' **← {**r**^*j *^: *j *∈ *J*^0 ^∪ *J*^+^}* * For (*j*^+^, *j*^-^) ∈ *J*^+ ^× *J*^- ^do	**Processing of constraints in a given order**
If and adjacent in **R **then	**Adjacency test**
* *end if * *end for *** *R **← **R'** End for	**Gaussian combination step**
**R **← **R \ **{ futile two-cycles } **R **← back-configuration of **R**	**Back-configuration**

As mentioned in the introduction section, the two most efficient algorithms for computing EMs available are the recently introduced null-space approach [24] and the Schuster algorithm [6], that we call "canonical basis approach" (implemented, for example, in METATOOL [29] version 4.3 and *FluxAnalyzer *version 5.0 [30]). Both algorithms handle reversible reactions directly. A direct handling of reversible reactions, meaning without network reconfiguration, is feasible in each setting and has been described in the respective original articles. This requires adapted adjacency tests. However, it does not affect the overall strategy. For simplicity, we describe these algorithms with networks of irreversible reactions only (the issue of reversible reactions is discussed below). We are now able to see that the algorithms of Schuster and Wagner differ basically only in the chosen initialization for **R**.

##### The canonical basis approach (Schuster approach; CBA)

The matrix **I **represents *q *independent rows extracted from **A **= [**N**^*T *^-**N**^*T *^**I**]^*T *^and can thus be used for **A**_*q*_. The matrix **A**_*q*_^-1 ^= **I**^-1 ^= **I **gives the *q *extreme rays that obey to these *q *independent constraints and works as initialization of **R**.

The remaining constraints are 2*m *linear inequalities defined by **Nr **≥ **0 **and -**Nr **≥ **0**, i.e. *m *equalities: **Nr **= **0**. The processing of an equality constraint is done in a single pass by only keeping rays of *J*^0 ^instead of *J*^+ ^∪ *J*^0^. This is achieved by replacing the line **R' **← {**r**^*j *^: *j *∈ *J*^+ ^∪ *J*^0^} with **R' **← {**r**^*j *^: *j *∈ *J*^0^} in the part "Processing of constraints in a given order" in Table 1. Note that in the original Schuster algorithm the values of **Ar**^*j *^(required for the Gaussian combination step) are explicitly stored throughout the algorithm (in the left-hand side of the tableau [4]) and adapted after each iteration.

##### The null space approach (Wagner approach; NSA)

The idea there is to initialize **R **by a well-defined kernel (or null-space) matrix **K **of **N **with a particular structure (the transposed **K**^*T *^is in (reduced) row-echelon form):



which can be computed, for example, by the MATLAB command *null*(*N*,'*r*'). One can assume **N **to be of rank *m*, the opposite case being discussed below ("On redundancies and network compression"). This implies  to be of size *m *× (*q *- *m*) and the identity of size *q *- *m*. This structure is obtained by allowing a reordering of the rows of **K**, i.e. of the reaction indices. Without losing generality, one can assume that the reactions corresponding to the block **I **are indexed from 1 to *q *- *m*. Consider the (*q *+ *m*) × *q *matrix . For all **x **in *P*(**A**_*q *+ *m*_), there is some vector **λ **≥ **0 ** such that **x **= **Kλ**. Reciprocally, for all **λ **≥ **0**, the vector **x **= **Kλ **lies in *P*(**A**_*q *+ *m*_). Thus (**A**_*q *+ *m*_, **K**) is a DD pair. Since **K **is a kernel matrix, its columns are independent vectors therefore (**A**_*q *+ *m*_, **K**) is a minimal DD pair. **K **as defined in (8) works as initial value for **R**. Hence, the initialization in this setup delivers directly *k *= *q *+ *m *solved constraints.

The remaining constraints are *m *linear inequalities defined by *r*_*i *_≥ 0, *i *= *q *- *m *+ 1...*q*. The Gaussian elimination step simplifies too



The right hand-side is practically a positive combination of the two vectors  and , because  is positive and  negative due to the definitions of *J*^+ ^and *J*^-^.

##### Adjacency tests

Here we give explicitly the adjacency test in the case of reconfigured networks for each setup. Variants handling reversible reactions directly were introduced for CBA and NSA. They lead in general to more complex algorithmic steps for a little (at most 2-fold) memory gain.

The test is used when processing the constraint *k *+ 1 to check whether two extreme rays **r **and **r' **of the cone *P*(**A**_*k*_) are adjacent. The adjacency test is based on Definition 2(b). Note that for a given extreme ray **r **of the cone *P*(**A**_*k*_), the considered zero set *Z*(**r**) is defined over the *k *constraints **A**_*k*_.

*CBA*: As mentioned above, in a CBA setup, equality constraints are solved within a single iteration. After the *l*-th iteration step, *k *= *q *+ 2*l *constraints are processed, therefore . The last 2*l *constraints are satisfied with equality for all computed rays. We denote by *Z*_*u*_(**v**) the Zero set of a vector **v **over the *u *first constraints. Here, with *u *= *q *it matches to the set of non-participating reactions in **v**. The adjacency test is then equivalent to the search of a third extreme ray **r" **such that *Z*_*q*_(**r**) ∩ *Z*_*q*_(**r'**) ⊆ *Z*_*q*_(**r"**). If such an **r" **exists, then **r **and **r' **are not adjacent.

*NSA*: After the *l*-th iteration step in an NSA setup, *k *= *q *+ *m *+ *l *constraints including *p *= *q *- *m *+ *l *sign constraints are processed. Thus . The last 2*m *constraints are satisfied with equality for all computed rays. Therefore, the adjacency test is then equivalent to the search of a third extreme ray **r" **such that

*Z*_*p*_(**r**) ∩ *Z*_*p*_(**r'**) ⊆ *Z*_*p*_(**r"**)     (10).

Thus, for NSA we only have to check the first *p*(*q *- *m *≤ *p *≤ *q*) elements of the rays, in contrast to all *q *elements for CBA. This is one reason behind the relative velocity of NSA compared to CBA.

#### On redundancies and network compression

It is common practice to reduce the problem of extreme ray enumeration by restricting the input set to the set of irredundant constraints [25]. Although the general problem of extreme ray enumeration is non-polynomial, the reduction into irredundant constraints is equivalent to linear programming and therefore of polynomial complexity. To our best knowledge, this important pre-processing has never been spelled out explicitly in the context of EM computation. However some network simplification steps have been proposed earlier [4,30] that deeply relate to the notion of redundancy removal. These simplifications include three heuristics that reduce the size of the original stoichiometric matrix **N **and thus the input size of the problem: the detection of conservation relations, of strictly detailed balanced reactions and of enzyme subsets.

**Figure 3:**
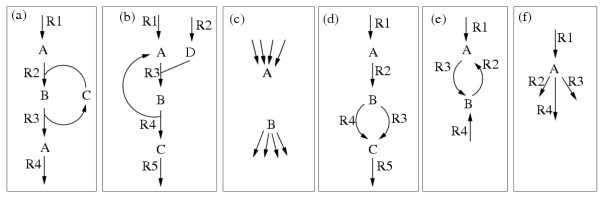
**Small example networks illustrating redundancies. **For explanations see text.

Conservation relations of metabolites are captured as linear dependencies between rows of the stoichiometry matrix **N **(thus, in the left null-space of **N**; [31]). This implies that some of the equality constraints in **Nr **= **0 **are linearly dependent. Satisfying a maximal linearly independent subset of these equations suffices to satisfy all equations. Therefore the problem can be reduced to , where  is the reduced stoichiometry matrix. For example, in Figure 3a, metabolites B and C build up one conservation relation and thus one of these metabolites can be removed. Note that conservation relations need not to be considered explicitly in the null-space approach since their removal does not affect the computed null-space matrix.

Conservation relations only consider redundancies among the equalities. The general approach handles also inequality constraints. Strictly detailed balanced reactions [32] and enzyme subsets [29] are particular cases of such redundancies. Strictly detailed balanced reactions are reactions with null flux at any steady-state. Many of them can be identified as null row vectors of **K**, the kernel matrix of **N**, and can be eliminated from the system. A non-trivial example is shown in Figure 3b, where R1 is strictly detailed balanced and would be detected by using the kernel matrix. However, there may be further reactions with a fixed zero-flux in steady state that cannot be identified by **K**. Some of those can be found by a simple analysis of **N**. For example, all the uni-directional reactions pointing into an internal sink (or emanating from a source) are certainly not participating in any steady-state flux (Figure 3c).

An enzyme subsets is defined as a group of reactions with relative constant flux ratio at steady state. Many of them can be identified as row vectors of **K **differing only in a scalar factor *α*. Reactions R1, R2 and R5 in Figure 3d would represent one enzyme subset. Assume one works on the reconfigured network and reactions R1 and R2 are members of the same enzyme subset. Thus, at steady state, we have for the respective rates *r*_1 _= *α *· *r*_2_. If *α *> 0, the constraints *r*_1 _≥ 0 and *r*_2 _≥ 0 are redundant, *r*_1 _≥ 0 being sufficient. In that case the practice is to lump both reactions into one lowering the number of reactions (and often also of the metabolites). If *α *< 0, the constraints *r*_1 _≥ 0 and *r*_2 _≥ 0 imply *r*_1 _= *r*_2 _= 0, hence, a special case of strictly detailed balanced reactions. In this case we say that the reactions contradict each other. Both reactions are not used and can be eliminated from the system as reactions R1 and R4 in Figure 3e.

**Figure 5:**
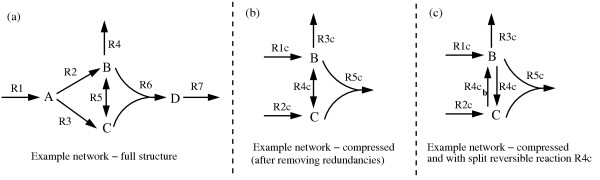
**Example network**. Full structure (a), compressed structure (b) and compressed structure with split reversible reaction R4c (c).

We identified another kind of redundancies. We call a metabolite M *uniquely produced *(respectively *consumed*) if only one single reaction, say *i*, can produce (respectively consume) M for several consuming (respectively producing) it (see Figure 3f). In that case, balancing metabolite M at steady-state implies that *r*_*i *_is always non-zero whenever the other reactions connected to M are active. We can therefore lump each reaction consuming (respectively producing) M with reaction *i *and remove metabolite M, decreasing the dimension of the problem further (see also the example in Figure 5 which is discussed below). Note that some enzyme subsets and strictly detailed balanced reactions can be seen as special cases of this type of redundancy.

Elimination of redundancies and network compression should be done in a pre-processing step leading to a compressed network structure. Thereby, it is important to detect and remove such redundancies *iteratively *until no further redundancy can be found. A MATLAB function *compressSMat *which removes all redundancies discussed above in an iterative fashion can be obtained from the corresponding author. After the computation of EMs, lumped reactions can be expanded to their single components.

There is a general approach for identifying redundancies in a set of linear constraints that uses linear programming, for example with the software *redund *distributed together with the software *lrs *[33]. This approach does not require any iterative process, but only identify redundant inequalities. Rows of **A **can be eliminated but no consequent column-wise reduction is done. Therefore, a simple redundancy removal is not as powerful as the accompanying network compressions presented above. The method however has the advantage to be systematic and might lead in the future to further network simplifications not yet identified.

### The binary approach

#### General idea

Using the reconfigured network with only irreversible reactions we have shown that the most important algorithms for EM computation belong to the same general framework. However, the original algorithms from Schuster and Wagner operate directly on the original network without splitting reversible reactions. At a first look, this seems to be more efficient since the dimension (number of reactions) is lower, decreasing seemingly also the memory requirement and the costs for adjacency tests. However, using the reconfigured network *S' *offers great simplifications. First, as already mentioned in an earlier section, the adjacency tests are easier to handle. The most important advantage, however, is the following. For the CBA in *S' *it follows that all non-zero elements of a ray **r**^*k *^will be retained if a new ray is obtained by combining **r**^*k *^with another (adjacent) ray because only positive combinations of rays are performed. The same holds for the NSA with respect to the *p *already processed inequality (irreversibility) constraints. This is of great importance since the adjacency test requires the information on zero/non-zero places in the rays only.

We illustrate this idea for NSA because this approach turned out to be more efficient than CBA. We assume that **N **has full rank *m*, i.e. there is no conservation relation. In this section, all variables correspond again to the network with split reversible reactions.

As described above, for an initialization of **R **we use a kernel matrix **K **of **N **having form (8):



Note that we use here the transposed representation of the tableau compared to Wagner's original article [24]. Since by eq. (9) only positive column combinations are performed during the algorithm, no negative number can show up in the upper part (consisting of *q *- *m *rows (reactions)) during the next iterations. The first row to be processed now is *p *= *q *- *m *+ 1. Using the general algorithmic scheme provided above all rays with non-negative entries at row *p *are retained and all negative entries can be combined with positive ones that are adjacent to them to obtain a zero at position *p*.

Assuming that the procession of the *p*-th row leads to a collection of *t *rays, we have:



The upper part, **R**_1_, contains the *p *processed rows which only contain non-negative values. Again, positive combinations of rays performed during the next iterations lead in the upper part to sums of non-negative numbers. Hence, it is easy to keep track of the zeroes in the upper part **R**_1 _by the use of bit masks. After the procession of the *p*-th inequality constraint the *p*-th row (i.e. the first row of **R**_2_) can be transformed to its binary representation and moved from **R**_2 _to **R**_1_. Using a binary representation for **R**_1 _has many advantages:

(i) For the next row *p *+ 1 to be processed we have to perform the adjacency test for pairs of vectors , . This test only requires the first *p *elements of these rays (see (10)), hence, exactly the columns of **R**_1_. Test (10) can then be written as a simple (and fast) bit operation. Two distinct vectors ,  are adjacent if and only if for all vectors **r**^*k *^distinct from  and , it holds:



( taken from **R**_1_; **r**_1...*p *_denotes the first *p *elements of **r**). Of course, the identical terms in the parentheses are computed only once.

(ii) Combination step of two adjacent rays (eq. (9)) reduces for the part in **R**_1 _to a simple OR operation, which is already computed for (13). The other (real number) components of the two rays (contained in **R**_2_) are combined as usual by eq. (9).

(iii) Bit operations as applied in **R**_1 _are not only fast, they are numerically exact in contrast to operations on real numbers.

(iv) The binary representation requires much less memory. Taking a typical 64-bit floating-point variable, storing **R**_1 _binary takes only 1.6 % of the memory needed for real numbers. Taking into account that in the worst case (all reactions reversible) the number of reactions in the reconfigured network is twice of that from the original one we still have a reduction in memory requirements of more than 96%. Note that **R**_2 _is empty at the end of the algorithm, hence, all EMs are then stored binary.

Bitmap representations of EMs have already been used in earlier implementations for accelerating the adjacency (elementarity) tests. However, binary tableaus had then been stored and updated *in parallel *to the full (real number) tableau of EMs which is not necessary here.

After the whole processing, EMs (extreme rays) are obtained for the reconfigured network *S' *as binary vectors. Binary patterns of EMs are completely sufficient for many applications of EMs (see discussion). However, a well-known lemma ([25] for example) ensures that this information is also sufficient to retrieve the real values up to a positive scalar:

Lemma 2

*In a d-dimensional Euclidean space, let ***r ***be a ray of the pointed polyhedral cone *P(**A**). *The following statements are equivalent*:

(a) **r ***is an extreme ray of *P(**A**)

(b) *rank*(**A**_*Z*(*r*)_) = *d *- 1

Each obtained binary vector provides the zero set *Z*_***q***_(**e**) and its complement the reaction set *R*(**e**) of an EM **e **in the reconfigured network *S'*. Lemma 2 says that the equation  and therefore

**N**_*R*(**e**)_**e**_*R*(**e**) _= **0 **    (14)

admit a one-dimensional solution space, i.e. the dimension of the null space of N_*R*(**e**) _is 1. **N**_*R*(**e**) _denotes the *m *× |*R*(**e**)| sub-matrix of **N **containing all those reactions (columns) of **N **which are involved in **e**. Solving the homogeneous linear system (14) gives a vector that can be normalized and properly oriented for example by dividing it by the value on its first participating reaction (see the example below). The reconstruction process reflects the fact that an EM is – up to a scalar – determined by its participating reactions.

In a second post-processing step, we transform the (real number) EMs of *S' *back into their representation in the original network *S *by using the rules given before Theorem 1. Note that it is also possible to transform first the binary EMs from *S' *into the binary EMs of *S *and then to reconstruct the real numbers (by using eq. (14) for the stoichiometric matrix of the non-reconfigured network *S*; see pseudo-code). In both cases, if the original network had been compressed during pre-processing, the EMs can finally be expanded to their corresponding modes in the uncompressed network.

#### Pseudo-code of the binary (null-space) approach

**Figure 4:**
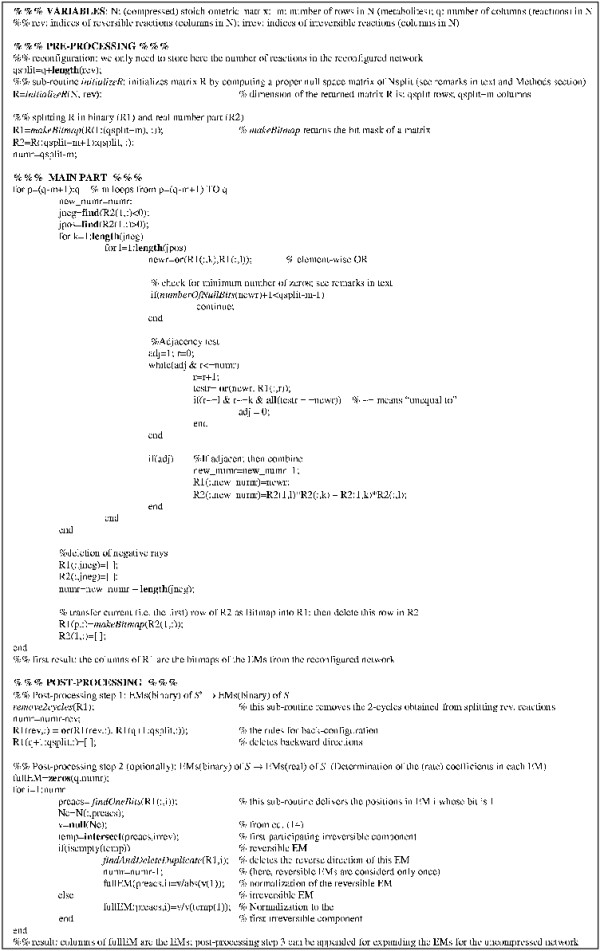
Pseudo-code: Core algorithm for computing elementary modes with the binary approach.

Using the results of the previous sections we are now able to give a pseudo-code of the binary (null-space) approach (Figure 4). The code follows MATLAB style, which provides a convenient and comprehensible notation for operations on vectors and matrices. We use several native MATLAB routines (written in bold). For concision, we also make the use of some other routines (indicated in italic). The code of the latter routines is not given here explicitly but their names and accompanying comments should allow the reader to implement them. For readers not familiar with MATLAB notation we give in the Methods section some basic explanations which should suffice for understanding the pseudo-code.

Note that the pseudo-code in Figure 4 is not given in its computationally most efficient form. It should just present the basic structure of the algorithm. There are two important issues in the algorithm we still have to discuss.

##### Minimal number of zeros in extreme rays (maximal pathway length)

In the null-space approach, the *m *equality constraints are always solved for each ray during the procession of sign constraints. Since any ray satisfies by Lemma 2 at least a total of *q *- 1 constraints, this implies that at least *q*-1-*m *sign restrictions are solved by equality. Hence each ray contains at least *q*-1-*m *zero-places. This fact can be used as a shortcut when checking the adjacency of two rays (see pseudo-code). At the end of the algorithm, it follows that the maximal pathway length |*R*(**e**)|_max_, that is the maximal number of involved reactions in an EM, reads (cf. [7]):

|*R*(**e**)|_max _= *q *- (*q *- *m *- 1) = *m *+ 1     (15)

##### Initialization of R

As for the non-reconfigured network, the initialization of **R **for the reconfigured network can be done with a null space matrix **K' **of **N' **having the special structure (8). Several of such kernel matrices may exist. We are interested in such a one that contains as many zeros as possible because the number of zeros in the starting tableau **R **has great impact on the number of ray combinations to be performed. For this purpose, it can be exploited that very sparse vectors of the null space of **N' **(not contained in the null space of **N**) are known, namely the two-cycles emerging by splitting up reversible reactions. We detail in Method section a technique that incorporates as many two-cycles as possible into **K **to construct **K'**.

#### Simple example

This section is devoted to illustrate our binary approach for computing elementary modes. Figure 5(a) shows a simple example network consisting of four metabolites (A,B,C,D) and 7 reactions (R1...R7), whereof R5 is reversible. The stoichiometric matrix **N **of this network reads accordingly:



Using our rules for removing redundancies, this network can be compressed as depicted in Figure 5(b). Metabolite A is uniquely produced, hence, R1 and R2 can be combined to R1c and reactions R1 and R3 are lumped into R2c. R3c and R4c correspond to the original reactions R4 and R5, respectively. Finally, R6 and R7 are enzyme subsets and are combined to R5c. Metabolites A and D can be removed, since they do not occur in any reaction anymore. Thus, the network dimension could be reduced by two metabolites and two reactions. The stoichiometric matrix **N**_*C *_of the compressed system reads:



From this compressed network, we can compute a null space matrix having structure (8), here even without permuting rows (reactions):



**K**_*C *_would be the starting tableau in the original null-space approach. Applying our binary approach we have now to split the (only) reversible reaction R4c in the compressed network (Figure 5(c)). This results in the stoichiometric matrix **N**_**C**_', where R4c_b _denotes the additionally introduced column of the backward direction of R4c:



Now we need to determine a null space matrix **K**_*C*_' of **N**_*C*_', if possible in the sparse form as in eq. (M1) (Methods section). **K**_*C *_– as given in (18) – contains only irreversible reactions in the identity sub-matrix. Therefore, without further rearrangements, we can already use it to construct **K**_*C*_' as described in the Methods section. We introduce an additional row in the identity sub-matrix of **K**_*C *_(corresponding to R4c_b_) and an additional column representing the two-cycle from the split reversible reaction R4c:



**K**_*C*_' is now a proper initialization for the **R **tableau according to (11). The first four rows (in the identity sub-matrix) can be seen as already completed, we therefore denote the starting tableau as **R**^4^. According to (12) we can divide **R**^4 ^into a binary (a non-zero entry is demarked by "×") and unprocessed real number part:



We proceed now with the 5-th row (R4c). All columns with non-negative entries in R4c are retained (columns 1 and 4). Columns 2 and 3 have a negative entry at position R4c and are therefore combined with 1 and 4 to obtain a zero at position R4c. In the binary sub-tableau, the combination step is a simple OR operation. Thereby, using the obtained binary patterns, the adjacency test (13) must be performed for each pair of combined columns. Here, all 4 possible pairs are adjacent. Accordingly, after completing row 5, tableau **R**^5 ^has 6 columns and reads:



Now we have already reached the last iteration step where R5c – the last row in real number format – is processed. Columns 1–5 are retained and column 6 is combined with columns 1,3 and 5. However, the column pairs (1,6) and (3,6) are not pairs of adjacent rays. This can be detected in two alternative ways. The usual way is that both column pairs violate condition (13) because of column 4. The second and quicker way is to observe that the minimal number of zeros in this network is 3 (*q'*-*m*-1 = 6-2-1) and that their respective combinations would give columns with only 2 zeros. These combinations are therefore not included in the tableau. We obtain:



Tableau **R**^6 ^is the binary representation of the EMs (extreme rays) from the split compressed network. Now, the post-processing begins. First, we remove the spurious 2-cycle (second column in **R**^6^) raised by splitting R4c. Then, rows R4c and R4c_b _are combined by an OR operation and row R4c_b _is dropped. Note, if a completely reversible elementary mode exists in the non-split network, it would lead to two EMs – one for each direction – in the split network. In such a case, either both are kept or only one, then marked as reversible EM. We have now obtained the 5 EMs of the compressed network as binary vectors:



Here, it is easy to reconstruct the real numbers of the EMs from their binary patterns. For illustrating the general case, we reconstruct the first mode **e**^1 ^using eq. (14):



The dimension of the null space of , hence of the solution space of eq. (25) is 1 (as it is for all EMs). A scalable solution vector is (2,1,1)^T^, normalizing to the first component yields the unique solution (1,0.5,0.5)^T^. Thus, the first EM in the compressed network is **e**^1 ^= (1,0,0,0.5,0.5)^*T*^. Reminding that we lumped the original reactions R1 and R2 into R1c and R6 and R7 into R5c, we can finally reconstruct the original elementary mode from the uncompressed network, that is *R*1 + *R*2 + 0.5 × *R*5 + 0.5 × *R*6 + 0.5 × *R*7.

#### Results from real networks

We implemented the binary null-space approach (binary NSA) in MATLAB (Mathworks Inc.) and incorporated it into the *FluxAnalyzer *[30,34]. The function includes a pre-processing step where the network is compressed as described. Some sub-routines of the algorithm are performed by compiled C-code (via MATLAB MEX interface), since this proved to accelerate the implementation drastically. In order to check the capabilities of our algorithm we computed the elementary modes in realistic and large metabolic networks. The three networks (S1-S3) considered here are variants from a model of the central metabolism of *Escherichia coli *investigated originally in [11,23]. For considering networks with different complexities we inserted an increasing number of substrate uptake or/and product excretion (pseudo) reactions, which increase the number of EMs much faster than the insertion of internal reactions. For a (rough) comparison with the original NSA we used the program *coverN *(developed by Clemens Wagner and co-workers; available upon request from clemens.wagner@pki.unibe.ch, which is also implemented in MATLAB and uses external C-files for some sub-routines. The original as well as the binary CBA algorithm proved to be slower than both methods of NSA (not shown).

Table 2 summarizes the computations. As a first result, it can be noted that redundancy removal and network compression during pre-processing results in much smaller networks. Note that the dimensions of the compressed networks of S1 and S2 are even lower than given in [23] due to the additional removal of uniquely produced/consumed metabolites. A lower number of reactions reduces the dimension of the null-space (hence, the number of iterations) and, in particular, the effort for adjacency tests. Generally, the proportion of the pre-processing on the overall computation time is negligible.

**Table 2:** Computations of elementary modes in a realistic metabolic network (central metabolism of *Escherichia coli*). Computations were performed on a typical PC with AMD Athlon XP 3000 + CPU and 1 GB RAM. Abbreviations: Form = formiate, Ac = acetate, Glc = glucose, Succ = succinate, Asp = aspartate, Glyc = glycerol, Eth = ethanol, Lac = lactate, CO2 = carbon dioxide.

	**S1**		**S2**		**S3**	
**substrates**	Glc		Glc, **Succ**, **Glyc**, **Ac**		Glc, Succ, Glyc, Ac, **Asp**	
**products**	Ac, Form, Eth, Lac, CO2		Ac, Form, Eth, Lac, CO2		Ac, Form, Eth, Lac, CO2, **Succ**	
**#reactions (q)** **# metabolites (m)**	106 (28 reversible) 89		110 (28 reversible) 89		112 (28 reversible) 89	
**compressed network:** **# reactions ** **# metabolites**	42 (17 reversible) 25		47 (17 reversible) 26		51 (17 reversible) 28	
**final number of elementary modes**	27,100		507,632		2,450,787	
	**binary NSA**	**NSA**	**binary NSA**	**NSA**	**binary NSA**	**NSA**
**computation time**	0.16 min (9.63 sec)	0.54 min (32.20 sec)	51.20 min	116.77 min	1546 min (25.78 h)	not finished
**back transformation**	0.13 min (7.97 sec)		2.57 min		13 min	
**total computation time**	0.29 min (17.60 sec)	0.54 min (32.20 sec)	53.77 min	116.77 min	1559 min (25.98 h)	

Comparing the required computation times, the binary NSA seems to be slightly faster than the original NSA. This observation should not be considered as a general result, since we cannot exclude that there are different potentials in optimizing the source code of *coverN *and in *FluxAnalyzer*, respectively. Besides, different row orders in the starting tableau can generally result in different computation times. However, it seems that the original and the binary NSA are comparable with respect to computation time. The adjacency tests in the binary null-space approach need to consider more elements (due to the split of reversible reactions) but are simpler to perform because preliminary modes from a previous iteration cannot lose their elementary property. Note also that implementing the full algorithm in C (and not only parts of it as in *coverN *and *FluxAnalyzer*) might further accelerate the computation considerably.

Using a special null space matrix **K' **as initialization of **R **(as explained in the Methods section) contributes considerably to a reduced computational effort. We can estimate this by the total sum  over the number of candidates *P*_*i *_occurring in the tableau before iteration *i*. In S1, for example, . Computing instead an arbitrary null-space matrix **K' **for **N' **(e.g. via MATLAB *null *command) results in a more dense initialization for **R **and the naive initialization would lead to . The larger numbers of candidates increase the costs for adjacency tests and accordingly the running time drastically. This underlines that the success of the null-space approach (in its original or binary form) depends strongly on the initially chosen null space matrix.

Generally, computing the stoichiometric coefficients of the EMs from their binary patterns is in larger networks in low proportion to the overall computation time (S3: ca. 0.8%).

Whereas the computational demands seem to be comparable for both null-space approaches, the memory requirements for the binary NSA are much lower, in particular during the last iterations. For this reason, the 2.45 millions of EMs from network S3 could be computed on a typical PC, whereas the original NSA ends in the 26-th iteration step (from a total of 28) due to memory overflow.

## Discussion

Elementary modes are smallest functional sub-networks, which can be interpreted geometrically as extreme rays from a pointed convex cone (corresponding to the network with split reversible reactions). The computation of extreme rays has been intensively studied by the polyhedral computation community and we think that the metabolic community can benefit from it. We shall also mention another abstraction of elementary modes within the framework of *matroid *theory [35]. In an *oriented vector matroid*, the elementary modes correspond to the *positive circuits *(or *positive cycles*), which are minimal dependent sets. In fact, an elementary mode is a minimal linearly dependent set of the column vectors of the stoichiometric matrix (in the reconfigured network with only non-negative coefficients). This has been mentioned only rarely so far [36]. Matroid theory could be a source for new theoretical investigations on elementary modes and could lead to further improvements in the computation procedure as well as to new applications in the sense of metabolic pathway analysis.

Adjacent extreme rays can also be detected by an algebraic characterization that completes Definition 2 [25]:

(*c*) **r ***and ***r' ***are extreme rays and the rank of the matrix *A_*Z*(**r**) ∩ *Z*(**r'**) _*is *d-2

In practical cases the characterization of adjacency is mostly computed in its combinatorial form than its algebraic one [25]. However, improvements could be done by using both characterizations. In fact, the test on EM length done before the actual adjacency test in our MATLAB pseudo-code is a consequence of the algebraic test. A striking feature of the algebraic test is that it only requires access to the two rays tested for adjacency (**r **and **r'**) and to the fixed size matrix **A**, in practice to the stoichiometry matrix. In comparison, the combinatorial test implies a loop over all other rays (**r"**). Therefore, the algebraic test could be suited for distributed computing.

Some theoretical issues of the combinatorial complexity of EMs were discussed in [23]. An upper bound *B *for the number of EMs is (reversible modes are counted only once):



Assuming that no conservation relations occur in the stoichiometric matrix, we obtain:



Note that *q *and *m *should be taken from the non-split, compressed network to obtain the lowest upper bound. In larger, realistic networks, even if compressed, the values for *B *explode quickly. Fortunately, the actual number of modes in real networks proved to be much smaller than the boundary (cf. *B *≈ 2.54 · 10^11 ^for S1 in Table 2), although it grows also exponentially. One reason is that many routes are not admissible due to violation of the sign restrictions. Another reason is the low connectivity of many metabolites leading to sparse stoichiometric matrices.

**Figure 6:**
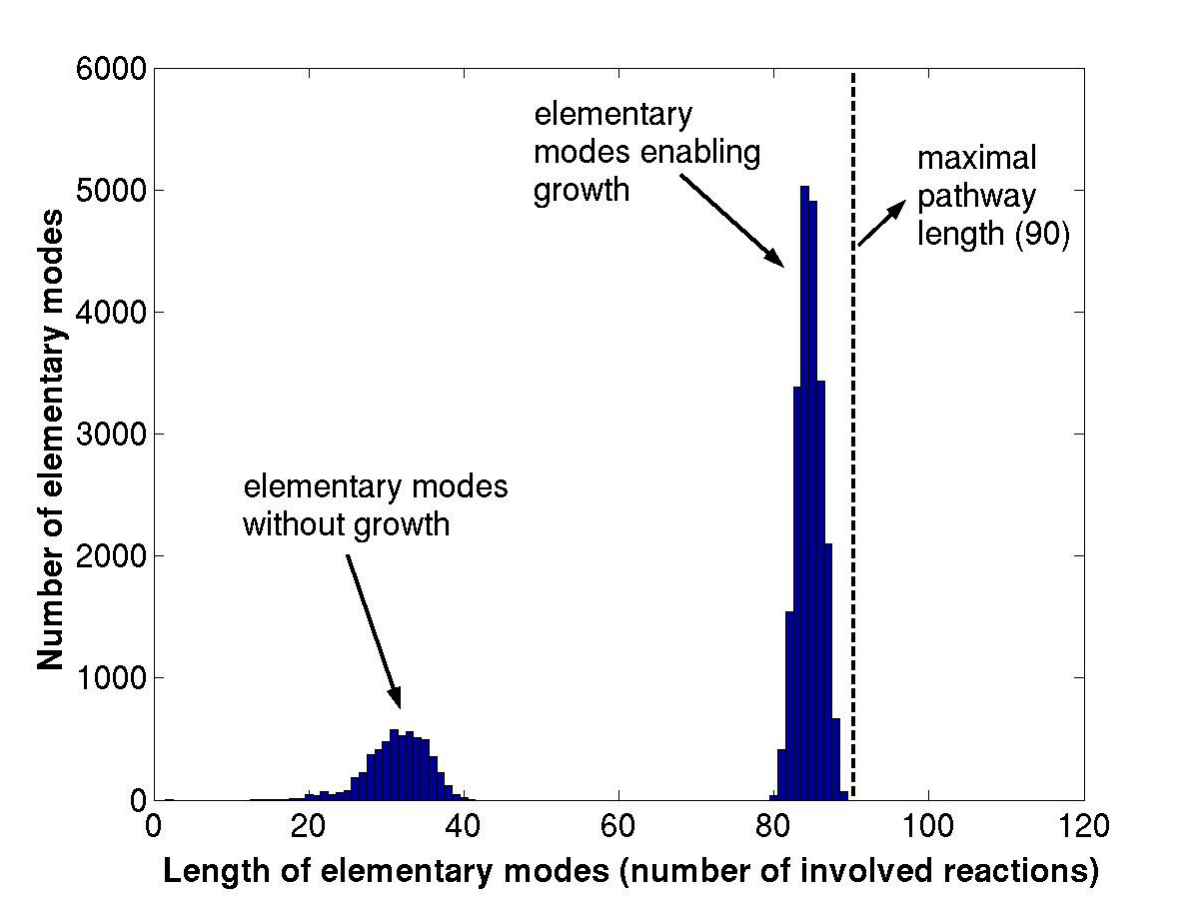
**Pathway length distribution in elementary modes of *E. coli. ***(Substrate: glucose; network S1 in Table 2).

A third reason is related to short pathway length. The upper bound reflects the case where all EMs have maximal pathway length |*R*(**e**)|_max _which is, by eq. (15), *m *+ 1. However, many EMs, if not all, have a lower length immediately reducing the possible number of modes [23]. The pathway length distribution of the *E. coli *modes on glucose (network S1) is shown in Figure 6. The maximal length of an EM in the uncompressed network is *m *+ 1 = 89 + 1 = 90. Modes that are not involved in biomass synthesis, in particular, are much smaller. In terms of linear algebra this means that there exist vector sets *W *containing fewer than *m *+ 1 column vectors of **N **that are linearly dependent. In polyhedral computation this phenomenon is known as *degeneracy*. Generally, degenerate systems may cause annoying difficulties and must be handled often differently to non-degenerate systems, albeit they reduce here the number of modes. The algorithms related to EM computation may be, in general, especially suited for computing extreme rays in such strongly degenerate systems, whereas other programs may be better suited for only weakly degenerate problems. For example, the software *lrs *[33] implements the so-called reverse search enumeration algorithm [37] that is polynomial for non-degenerate cases. Note that the new binary approach as introduced herein can easily be adapted for computing extreme rays of any pointed cone as given in eq. (4) and may therefore improve the performance of extreme ray computation in many other applications.

Albeit the general framework was formulated long time ago, the explicit introduction of the null-space approach was an important mile-stone in accelerating the computation of EMs. The binary null-space approach as introduced herein increases the efficiency of this approach also with respect to the memory requirements and enables now to compute EMs in networks significant larger as those investigated before. A simple computation gives the number of about 85 millions of EMs in a network of 100 (compressed) reactions that can be stored in 1 GB RAM (cf. compressed and reconfigured S3: *q' *= 51 + 17 = 68). Of course, only a fraction of this amount can be stored during the algorithm due to other (partially large) temporary variables. Besides, reactions that are not yet processed are still stored as real numbers. The amount *M *of memory required for storing *E *modes after the procession of *p *reactions (stored binary) is (assuming 64-bit real numbers)

*M *= *E *· (*p *+ 64 · (*q *- *p*)).     (28)

It depends on the evolution of the number of EMs during the algorithm where the maximal memory demand occurs. Generally, much larger networks can now be treated.

## Conclusions

The four main results of this work are: (i) showing the equivalence between extreme rays and elementary modes, (ii) showing that algorithms for computing elementary modes can be seen as variants of the double description method for computing extreme rays in pointed polyhedral cones, (iii) introduction of a general framework and of new methods for redundancy removal and network compression, (iv) introduction of the new binary approach for computing extreme rays and elementary modes.

The binary approach computes elementary modes as binary patterns of participating reactions that are sufficient to compute the respective stoichiometric coefficients in a post-processing step. For many applications – following the computation – it is even sufficient to operate on the binary patterns of EMs. Among all applications of EMs presented in the introduction section, only the identification of all pathways with optimal yield, the "control-effective fluxes", and the *α*-spectrum need the explicit (real number) coefficients, i.e. the reaction rates, in the EMs. Whenever needed, the explicit representation of an EM can be determined (possibly temporarily) from its binary pattern.

The binary approach decreases the memory demand up to 96% without loss of speed and without loss of information giving the most efficient method available for computing elementary modes to date. The limiting step in computing elementary modes has thus been shifted back to the computation time. Parallelization – as investigated within the traditional, not-binary, schema in [38] – might lead to a further acceleration bringing us again a step closer to the complete set of EMs in genome-scale metabolic networks.

## Methods

### Proof of Theorem 1

We prove first that each case a) and b) defines EMs of *S'*. Let **e' **be a flux vector defined by either case a) or b). Clearly **N'e' **= **0 **and **e' **≥ **0**. In case b) **e' **is not elementary only if the single forward or backward reaction balances all internal metabolites, i.e. if the reaction includes not any internal species. We can safely exclude this pathologic case by considering that **N **does not contain a null column. Therefore, **e' **is elementary. In case a), assume **e' **is not elementary, i.e. there exists a non-null flux vector **x' **of *S' *not equivalent to **e' **such that **x' **≥ **0**, **N'x' **= **0 **and *R*(**x'**) ⊆ *R*(**e'**). By definition of the reconfiguration, for each *i *∈ *Rev*, at least one among *e'*_(*i*,+1) _or *e'*_(*i*,-1) _equals zero and this holds consequently also for **x'**. Thus one can define **e **and **x**, the back-configurations of **e' **and **x'**. Now, by definition, **e **is an EM of S and is not equivalent to **x**, **Nx **= **0**, *x*_*i *_≥ 0_*i *_for *i *∈ *Irrev *and *R*(**x**) ⊆ *R*(**e**), a contradiction.

Hence each case a) and b) defines EMs of S'. We prove now that there is no other case. Assume there exists **e' **neither defined by a) nor b), such that **e' **≥ **0**, **N'e' **= **0 **and **e' **elementary. For each *i *∈ *Rev *at least one among *e'*_(*i*,+1) _and *e'*_(*i*,-1) _equals zero (otherwise the two-cycle defined on reaction *i *would satisfy the constraints and involve only a subset of the reactions of **e'**). Thus the back-configuration **e **of **e' **can be defined. By definition, **e **is not an EM of S. There exists **x **not equivalent to **e **such that **Nx **= **0**, *x*_*i *_≥ 0_*i *_for *i *∈ *Irrev *and *R*(**x**) ⊆ *R*(**e**). The reconfiguration **x' **of **x **is such that **x' **is not equivalent to **e'**, **x' **≥ **0**, **N'x' **= **0 **and *R*(**x'**) ⊆ *R*(**e'**), a contradiction.

### Initialization of the **R **tableau in reconfigured networks

As in the case of non-reconfigured networks, we must initialize **R **in reconfigured networks as a null space matrix **K' **of **N' **having the special structure (8), i.e. . Several kernel matrices having this form can exist. Here we are interested in such a one that contains as many zeros as possible because the number of zeros in the starting tableau **R **has a great impact on the number of ray combinations to be performed. For this purpose, we can exploit the fact that we already know |*Rev*| many very sparse vectors of the null space of **N**', namely the two-cycles emerging by splitting up reversible reactions. Our goal is therefore to incorporate many (if possible all) of these vectors into **K **to obtain **K'**. For this purpose, we first compute the kernel matrix **K **of **N**. Then, by simple linear combinations of columns (analogous to the well-known computation of a row-echelon form of a matrix) and possibly by permutation of rows in **K**, we try to obtain , where only irreversible reactions (rows) are contained in the identity matrix **I**. If this is possible then we can easily include the backward directions of reversible reactions (as rows) and the two-cycles (as columns) into **K **yielding **K'**:



The first *q *- *m *columns in **K' **correspond to the original columns in **K**, but contain additionally zeros for the inserted backward reaction of originally reversible reactions. These columns are obviously linearly independent and are contained in the null space of **N'**. Sub-matrix  is a |*Rev*| × |*Rev*| identity matrix whose rows correspond to the backward directions of split reactions. Finally, **C **is a |*Rev*| × *m *sub-matrix which complements  in such a way that they represent together the two-cycles of the split reactions. (Thus, each column *c*_*i *_in **C **contains only zeros, except a unity at that row, which corresponds to the forward direction of the split reversible reaction *i*. See also the example network.) **I **and  yield together the new **I'**, whereas  and **C **represent together  of **K'**. Thus, **K' **contains *q *- *m *+ |*Rev*| linearly independent (basis) vectors of the null space of **N' **and is in form (8).

To our experience, in most realistic networks, a matrix **K' **as in (M1) can be found. Using instead an arbitrary **K' **can lead to a much larger computation effort because much more candidates are computed at an early state (see real network examples).

A further simple strategy avoiding that many rays are computed early is to sort the rows in  ascending with respect to the number of their non-zero entries.

In case it is not possible to arrange only irreversible reactions into the sub-matrix **I **of **K**, we can nevertheless find a matrix **K' **with the same basic structure as in (M1). However, for some originally reversible reactions, the forward (in **I**) *and *backward (in ) direction will then be contained in **I'**. For each of those, the two-cycle cannot be represented by **C **and  (because the row of the forward direction is contained in **I' **and not in **K'**) and another corresponding column in **C **has to be constructed. Assume a reversible reaction is contained as *j*-th row in **I**. Assume further that the inserted backward direction of this reaction corresponds to the *k*-th row of . For the *k*-th column **c**_*k *_of **C **we can then chose the *j*-th column  of  multiplied by -1, i.e. . Together with the *k*-th column in , this gives a null space vector of **N'**, which is linearly independent of the others and can therefore serve as basis vector in **K'**. The vector **c**_*k *_is now probably not that sparse. However, it enables us to retain the 2-cycles at least for those split reactions whose forward direction is not contained in **I**.

A MATLAB function *initializeR *that provides a proper initialization of **R **as described above (starting with the stoichiometric matrix **N **and the indices of the reversible reactions) can be obtained from the corresponding author.

### Short introduction into MATLAB notation

Numeric variables in MATLAB can be scalars, vectors or two-dimensional arrays (i.e. matrices). To be more precise, a scalar in MATLAB is actually a 1 × 1 array and a vector is a 1 × n or n × 1 array. Size and type of a variable are automatically declared (or changed) by assignments to it. The following examples illustrate how to assign or access values of variables:

• scalar: a = 1;

• b(3) = 5; the value 5 is assigned to the third element of (vector) *b*.

• c(1:3) = [5,8,9]; here, "1:3" expresses "from 1 to 3", thus, 5, 8 and 9 are assigned to the first three elements of vector *c*. It is also possible to use an array of integers to access the elements of a vector, e.g. a = [2,3,4]; b = [1,3]; c = a(b). Vector c reads then [2,4].

• mat(2,5) = 3; value 3 is assigned to the element in the second row and fifth column of matrix *mat*.

• mat1(3,:) = mat2(5,:); the values of the fifth row of matrix *mat1 *is copied into the third row of matrix *mat2*. Here, the colon operator ":" expresses "all elements of the respective dimension" (here: columns). Of course, it must be ensured that *mat1 *and *mat2 *have the same number of columns.

• a = mat(7,1:3); the first three elements of the seventh row of matrix *mat *are assigned to *a *which is now a 3-element vector.

• a= [17,34,39]; a(2)= []; deletes the second element of *a *and shifts all elements behind one position back, i.e. vector *a *reads now [17,39].

The pseudo-code given in Figure 4 in the main text uses several basic routines pre-defined in MATLAB (written in bold) :

• c = **length**(a); if *a *is a vector (as in all cases in the pseudo-code) then **length **returns the number of elements in *a*.

• c = **find**(a); if *a *is a vector (as in all cases in the pseudo-code) then **find **returns all positions in *a *which are not zero. Example: **find**([23,0,5,9,0]) returns (1, 3, 4).

• c = **or**(a,b) returns the result of the logical OR operation applied element-wise to *a *and *b*. *a *and *b *can be scalars, vectors or matrices and must have the same size. Example: if *a *= [1,0,29], *b *= [1,0,0] then **or**(*a*,*b*) returns [1,0,1]. In the pseudo-code, we use this routine exclusively for OR-operations of bit masks (arrays with only "ones" and "zeros").

• c = **zeros**(m,n) returns a matrix of size m × n filled with zeros.

• c = **null**(a) returns a null-space matrix of matrix a.

• c = **intersect**(a,b) returns the intersection of elements in vectors *a *and *b*.

• c = **all**(b) returns "1" if all entries in vector b are not zero and "0" otherwise.

## List of abbreviations

EM(s): Elementary Mode(s) also known as Elementary Flux Mode(s).

## Authors' contributions

Both authors contributed equally to this work, the starting idea of the binary approach coming from a discussion between them. JG mainly established the relationships between extreme ray and elementary modes computation. SK mainly devised and implemented the binary null-space algorithm. Both authors prepared the manuscript jointly.
